# Specialist medication monitoring and prescribing in primary care: case study of shared care agreements in Northern England, UK

**DOI:** 10.1136/bmjoq-2025-003491

**Published:** 2025-11-19

**Authors:** Matthew Cooper, Victoria Trotter, Annette Hand, Hamde Nazar

**Affiliations:** 1 Newcastle University Faculty of Medical Sciences, Newcastle upon Tyne, UK; 2 Brampton Medical Practice, Brampton, UK; 3 Northumbria University, Newcastle upon Tyne, UK; 4 Durham University, Stockton, UK

**Keywords:** GENERAL PRACTICE, Pharmacists, Quality improvement, Patient safety

## Abstract

**Introduction:**

Shared care agreements (SCAs) in the UK enable general practitioners (GPs) in primary care to take over the monitoring and prescribing of specialist medications for patients under agreed protocols. While SCAs are intended to improve access and continuity of care, concerns regarding their implementation and adherence to safety protocols persist. This study aims to explore the mechanisms, challenges and risks associated with SCAs, focusing on their impact on patient safety and primary care capacity.

**Methods:**

A case-study approach was employed to investigate the implementation of SCAs, incorporating mixed methods to provide a comprehensive understanding. Data triangulation included document analysis of policies, cross-sectional review of medication monitoring and prescribing practices across 37 GP practices, and key informant interviews with stakeholders. Logic and dark logic models were iteratively developed to map the intended and unintended outcomes of SCAs.

**Results:**

The monitoring and prescribing review revealed 32.3% of prescribed medications under SCAs lacked up-to-date monitoring data, with attention-deficit/hyperactivity disorder medications showing the highest rates of non-compliance. Interviews highlighted systemic challenges, including unclear responsibilities, inadequate patient involvement, fragmented communication between primary and secondary care, and insufficient integration of digital systems. These gaps contribute to patient safety risks, particularly for high-risk medications requiring stringent monitoring.

**Conclusions:**

SCAs hold potential for improving care continuity but face significant operational and systemic barriers that undermine their safety and effectiveness. Findings evidence the need for clearer role delineation, robust communication frameworks, enhanced patient engagement and integrated digital solutions. Policy-makers and healthcare leaders must address these challenges to ensure SCAs deliver on their promise of seamless, safe and sustainable care. Future research should focus on incorporating the perspectives of secondary care providers and pharmacists to develop more inclusive solutions.

**WHAT IS ALREADY KNOWN ON THIS TOPIC**

- Shared care agreements (SCAs) aim to transition the monitoring and prescribing of specialist medications from secondary to primary care. Shared care protocols must be patient-specific and clarify individual roles and responsibilities, medicine details, patient monitoring and circumstances where treatment should be modified or stopped. The agreement aims to ensure seamless prescribing and monitoring of medicines, enabling patients to receive care in an integrated manner.

**WHAT THIS STUDY ADDS**

- This study identifies critical gaps in the implementation of SCAs, such as inconsistent adherence to monitoring protocols and limited patient involvement.

**HOW THIS STUDY MIGHT AFFECT RESEARCH, PRACTICE OR POLICY**

- The study highlights the urgent need for reforms in shared care practices, emphasising clearer role definitions, better communication systems and improved patient engagement. These findings can inform policy changes, support primary care decision-making and guide future research to enhance the safety and effectiveness of SCAs.

## Introduction

 In conventional clinical practice in the UK, some conditions require the expertise and oversight of specialist practitioners. Such conditions would usually be diagnosed in specialist hospitals and/or clinics by consultants of this specialty, for example, clinical psychiatrists diagnosing bipolar disorder in mental health settings. Medication to manage these conditions would be initiated in these specialist environments, for example, lithium for bipolar disorder. Often, these medications require longitudinal monitoring following the initial loading dose period, for example, an annual physical health check (blood pressure, lipids, fasting blood glucose/HbA1c, ECG) for lithium. Results of such monitoring have the potential to influence onward prescribing or deprescribing of medication.

In the UK, a process is available when a specialist considers a patient’s condition to be stable or predictable, they can seek the agreement of a general practitioner (GP) in primary care to share the care of that patient.^1^ The established shared care agreement (SCA) is usually between a hospital or the specialist setting and the GP (although other primary care professionals may be involved), where the GP monitors and prescribes the specialist medication. Shared care protocols must be patient-specific and clearly outline individual roles and responsibilities, medication details, patient monitoring procedures and specific circumstances, such as the thresholds for results from monitoring and when treatment should be modified or stopped.^2^ The agreement aims to ensure seamless prescribing and monitoring of medicines, enabling patients to receive care in an integrated manner.^1^

The range of medications that can be considered for an SCA is diverse, often high-risk and has a range of monitoring requirements. There is currently no evidence to suggest that medications being prescribed through this process are well-managed or well-monitored in accordance with their specific requirements.^3^ Given the nature of the medications involved, there is a high potential for patient risk. The ongoing implementation and delivery of care via SCA is at risk due to the pressures on the healthcare system, but it is not clear what risk, or indeed reduction of risk, this presents to patient care and safety. There is a need to investigate and explain the current implementation and delivery of SCA concerning patient risk. It is important to find evidence to demonstrate if and how SCA might be working (or not) to ensure patients receive seamless care and continuity of medication access. Findings can be considered helpful in informing safer and sustainable strategic decision-making for GP practices.^4 5^

## Methods

### Research design

We used a case-study approach to better investigate and understand SCAs and how they are working (or not) in the complex context of the healthcare system. This approach is an established research design used to generate an in-depth, multifaceted understanding of a complex issue in its real-life context. This design allowed capturing information about ‘how’ an intervention or policy is being implemented and received on the ground, ‘what’ gaps or risks exist in its delivery or why implementation and delivery are happening as they are.^6^ Our case study is instrumental in design as we have captured data from different stakeholders, regions and data types to understand and explain what the ‘typical’ patient risk is with regards to shared care medication.

To triangulate the data, we used evidence from a regional review of prescribing and monitoring, document analysis of policy documents and agreements, and key informant interviews. In line with the case study approach, data were collected in different ways, approaching the same issue from different angles to help develop a holistic picture of the phenomenon. This integration of typically disparate sources of data offered a coherent interpretation that can otherwise be challenging to interrelate.

We used the scaffold of logic model development to bring together, make sense of and present our findings.^7 8^ The logic model was iteratively developed and reviewed through data collection and analysis to illustrate how and why the shared care medication pathway is being implemented and received. To create a logic model, data are coded to the main components: inputs (resources required), activities (actions carried out), outputs (direct products or services delivered) and outcomes (what you expect to change). Once mapped, data are arranged visually to show the logical links between each of the core components, along with any underlying assumptions (the mechanisms of change). While logic models examine the positives of an intervention, dark logic examines the negatives. We developed a ‘dark’ logic model to highlight the challenges, gaps and patient risks that exist in this package of care. Dark logic models aim to illustrate mechanisms by which an intervention hypothetically has adverse effects on the outcomes of interest (paradoxical effects). These models were developed by scrutinising models of intended change and their assumptions (eg, logic models) by actively hypothesising and, where possible, finding evidence to highlight paradoxical effects and harms.^9^ The benefit of dark logic, and the rationale for its application within this research, is that it can systematically identify plausible harm to recognise risk sooner, improve monitoring standards and provide a plausible mechanism to prevent harm.

### Mixed-methods approach

We adopted a convergent mixed-methods approach where data is being collected across various sources in different ways and integration of data occurs during analysis that is proceeding in an interactive manner.^10^

### Document analysis of SCA documentation

Document analysis was applied to provide a systematic method for reviewing the available literature on SCAs. Due to the limited research on SCAs (scoping searches retrieved fewer than fifteen articles), document analysis allowed for the triangulation of relevant information from policy documents, peer-reviewed literature and practice protocols to develop the logic models. The three stages (skimming, reading and interpretation) outlined by Bowne were used to extract relevant data to inform the development of the logic model.^11^ These three steps were followed sequentially, with relevant data extracted and refined following each step. Due to the small sample of documents available to analyse, the principle of information power was applied (indicating that the volume of information contained within the documents was sufficient to meet the researcher’s requirements in the development of a logic model).^12^

### Cross-sectional review of prescribing and monitoring of shared care medications

A review was undertaken to explore how SCAs were working in general practice. A group of general practices within the Northeast of England had expressed interest in better understanding the challenges around shared care at a regional strategic committee (Integrated Care Board (ICB)). Through an existing collaborator at the ICB, the group of practices was sent a proposal in March 2024 (online supplemental appendix 1) to gain information about:

What medicines were being prescribed by the general practices under shared care agreements?

What monitoring requirements were being adhered to for these medications as per the agreement specifications?

The general practices covered an area of 111.8 km^2^ and provided services to over 285 000 patients, where more than 74% of this population resided in areas of the lowest deprivation (IMD 1–5). The practices agreed to this proposal in May 2024 and provided an Excel spreadsheet of anonymised data across their 37 practices in August 2024.

Data were analysed per medication against the specific monitoring requirement to illustrate the level of adherence to shared care medication requirements.

### Key informant interviews

Key informant interviews were conducted to understand and explain how SCAs were implemented. Following the model of information power, this study was deemed to need a small number of participants due to fitting the following criteria:

- The study aim is narrow (ie, implementation and delivery of SCA).
- The combination of participants is highly specific to the study aim (ie, those involved in SCA implementation and delivery, including GPs and pharmacists).
- It is supported by established theory (ie, logic model development).
- The interview dialogue is strong (ie, the quality of information will be high and rich).
- The combination of participants is highly specific to the study aim (ie, those involved in SCA implementation and delivery, including GPs and pharmacists).

This rationale guided our approach to purposively select and invite at least two representatives from each level of the SCA context for an interview. We aimed to recruit participants to interview across the micro (eg, GP practitioners, specialist consultants, pharmacists), meso (eg, GP managers, practice partners) and macrolevels (eg, policy-makers and clinical directors). These were recruited via existing contacts, networks and collaborators rather than through random sampling. These individuals were known to have expertise and working experience with SCA and their medications.

A topic guide was used to support data collection. The topic guide was developed iteratively throughout the interviews and initially based on information extracted from the document analysis. The topic guide covered questions on their perspectives of the prescribing and monitoring data from the review; key resources, activities, outcomes and impact required to make SCA work, and the key barriers and facilitators to patient safety.

Individuals were emailed participant information sheets to describe the study and a consent form to complete to indicate their willingness to participate. Interviews were conducted by one of two researchers and recorded using Microsoft Teams employing the transcription functionality. Transcripts were downloaded and read while listening to the audio to correct and amend any discrepancies. Basic content analysis^13^ was employed across the transcripts to identify information about the elements of the logic model (ie, resources, activities, outcomes and impact). The logic models were reviewed and adapted iteratively as each transcript was analysed until no new amendments could be made. This was undertaken by one researcher and verified with the second researcher until a consensus was reached. General thematic analysis following the recognised six steps^14^ was then undertaken to investigate the key issues concerning patient safety.

Patients or the public were not involved in the design, or conduct, or reporting, or dissemination plans of this research.

## Results

### Document analysis

10 documents were included in the analysis (5 research papers and 4 policy and practice guidance documents). A summary of these documents is shown in table 1.

**Table 1:** The documents used in the documentary analysis

Author/organisation	Type of document	Main findings/guidance
Carrington and McAloon J.^15^	Research: mixed-methods study of GP doctors’ views of ADHD shared care prescribing	GPs provided many reasons for not accepting SCA for ADHD medication. This included concerns about the diagnosis, lack of availability or uptake of non-pharmacological therapy and poor perceptions of the onward patient monitoring.
Chana N, *et al*.^2^	Research: human factor methodology to explore the use of clinical decision support system to support the safety and quality of specialist drug prescribing	The current prescribing process of specialist medications is prone to errors, which have a high probability of occurrence. A Clinical Decision Support System to support GPs in the process is needed to reduce errors and increase patient safety.
Horne R, *et al*.^16^	Research: qualitative study of doctors’ views of prescribing specialist medications	GPs expressed dissatisfaction with SCA due to problems around clinical responsibility, ‘cost shifting’, availability of medicines and nature of prescribing relationship.
Duggan C, *et al*.^3^	Research: investigating the implementation of shared care agreements	‘Cost-shifting’ was a key concern triggering cynicism with SCAs. There was a lack of evidence about benefits to patients and implementation and distribution were erratic. There were recommendations to better involve healthcare professionals, improve communication systems and address system barriers.
Iqbal N, *et al*.^18^	Research: a systematic review of the role of GP pharmacists in supporting the implementation of shared care agreements	No studies were found that investigated the activities and interventions of pharmacists in primary care on SCA provision.
Nottinghamshire APC.^19^	Frequently asked questions: about shared care for patients and carers	Includes information about what is shared care; the roles and responsibilities of the specialist, GP and the patient and whether shared care is possible between a private care provider and a GP
Local Medical Committee Guidance, Wessex.^20^	Guidance: Understanding Shared Care–NHS, Right to Choose and Private Providers	Provides guidance to GP staff about the National Health Service providers, private providers, the nature of shared care agreements and some practical advice about delivery
British Medical Association.^21^	Guidance: General practice responsibility in responding to private healthcare	The guidance outlines that shared care with private providers is not recommended due to the general NHS constitution principle of keeping as clear a separation as possible between private and NHS care.
NHS England.^22^	Policy: Responsibility for prescribing between primary and secondary/tertiary care	Outlines the responsibilities and behaviours required to support safe prescribing under shared care

ADHD, attention-deficit/hyperactivity disorder; GP, general practitioner; SCA, shared care agreement.

**Figure 1:**
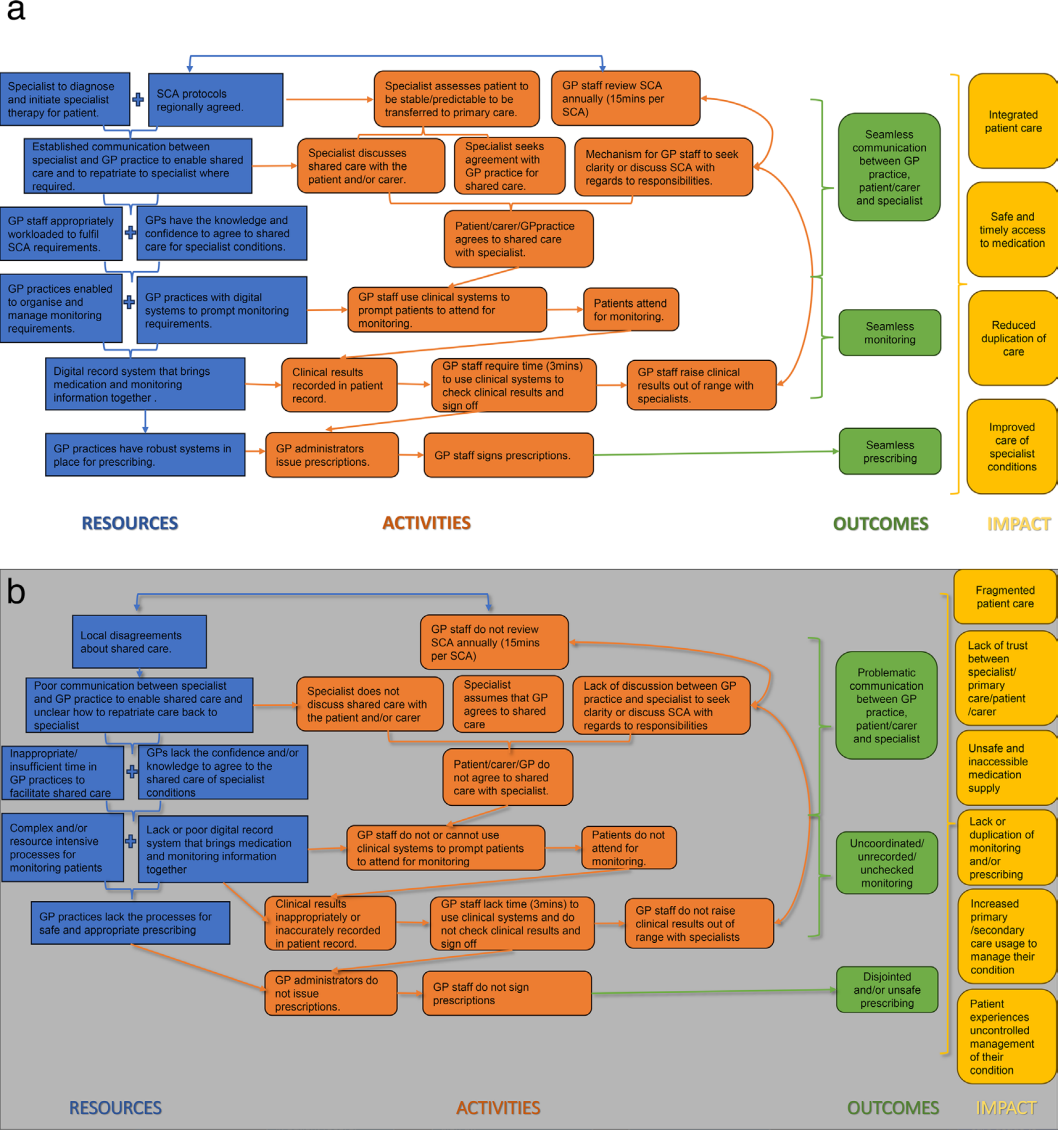
(a) Logic model to outline how shared care should work to deliver impact. (b) Dark logic model to highlight potential challenges, gaps and risks within the shared care model. GP, general practitioner; SCA, shared care agreement.

The initial logic and dark logic models developed from the information from these documents are shown in figure 1a,b.

### Review of prescribing and monitoring

The data demonstrated that 15 medications were being prescribed against SCAs across the practices. This totalled 2773 individual prescribing instances. 897 (32.3%) of these records showed monitoring data that was not up to date. (Full data about the monitoring requirements for each drug are in online supplemental appendix 2) More significantly, in 234 (8.4%) of these cases, the SCA medications had been prescribed. Medications being prescribed for attention-deficit/hyperactivity disorder (ADHD) (dexamphetamine, lisdexamphetamine, methylphenidate, atomoxetine) were those that had the highest out-of-date monitoring data. For two of the medications, there were no monitoring requirements indicated within the SCA (table 2).

**Table 2:** The shared care medications and details about the currency of the required monitoring

Medication	Number	Number of out-of-date monitoring	Number of medications prescribed with out-of-date monitoring
Azathioprine	151	68	16
Denosumab	82	45	3
Lithium	162	136	136
Hydroxychloroquine	501	Monitoring not indicated	
Sulphasalazine	339	Monitoring not indicated	
Leflunomide	81	20	3
Methotrexate	615	255	17
Dexamphetamine Lisdexamphetamine Methylphenidate Atomoxetine	670	326	195
Dronedarone	24	19	
Amiodarone	42	23	
Mycophenolate	72	18	
Mercaptopurine	34	7	

### Key informant interviews

**Figure 2:**
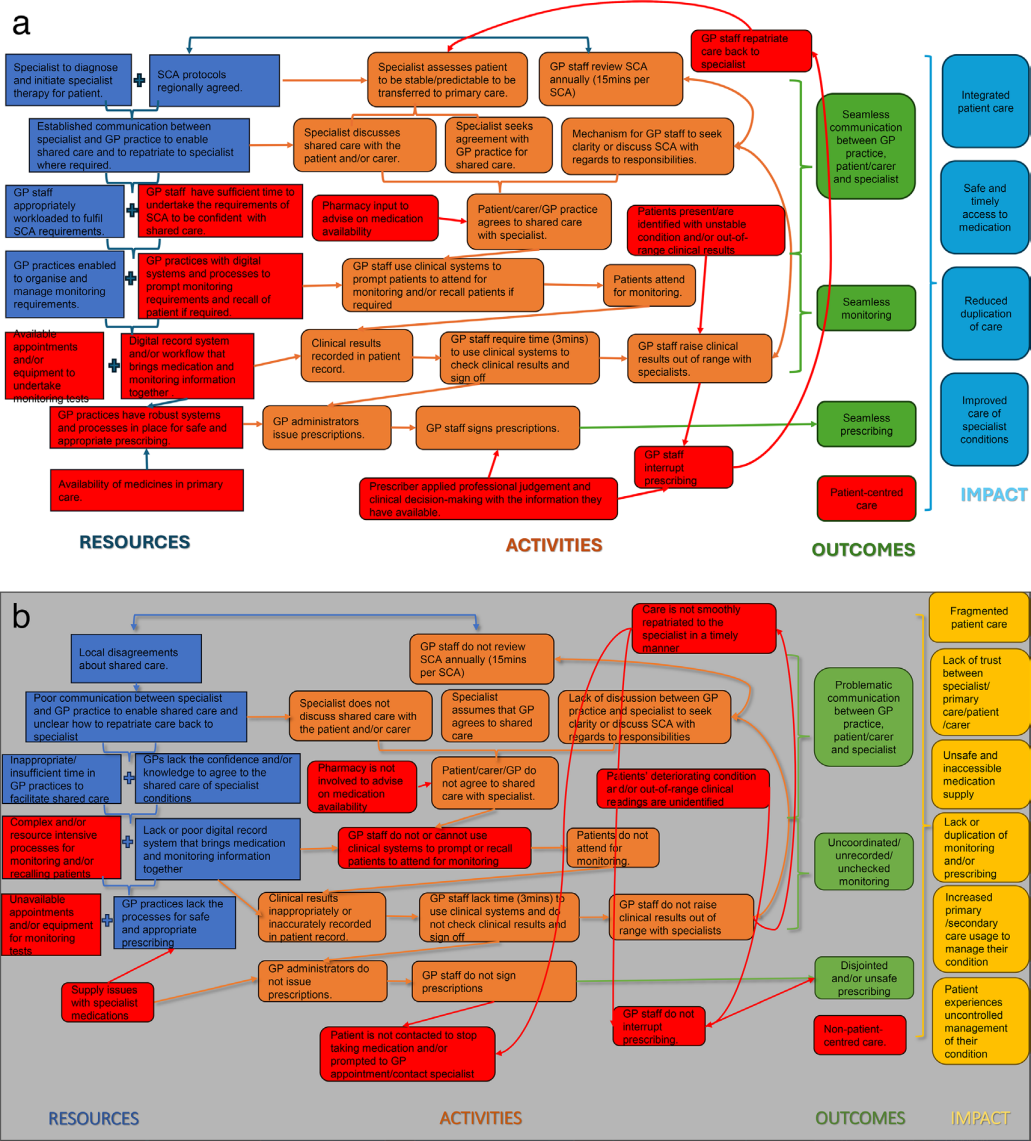
(a) Revised logic model where red indicates additional insights from key informants about how shared care should work to deliver impact. (b) Revised dark logic model where red indicates additional insight from key informants about challenges, gaps and risks within the shared care model.

Interviews were conducted on a one-to-one basis (HN and MC). Neither interviewer knew participants before the study, with one (MC) having no prior medication knowledge or topical insight. Eight key informants were recruited across the micro (n=3), meso (n=3) and macro (n=2) levels and interviewed. Job roles of participants included GPs, pharmacists, practice managers, regional representatives from policy and commissioning. Interviews took place between October and November 2024 and lasted between 45 and 62 min. The logic and dark logic models were refined and are presented in figure 2a,b. Interviewees raised several patient safety concerns associated with SCA (Illustrative quotes for the themes are included in online supplemental appendix 3).

### Absence of patient input and involvement in shared care

Patients are not consulted or included in the conversations when setting up shared care. This is perceived to lead to confusion for patients who will lack an appreciation of the processes behind shared care. It can undermine the perceived importance of monitoring requirements and have implications for the safe and appropriate prescribing of their medication. Interviewees alluded to the fact that this ignorance could lead to patient harm, through not attending monitoring appointments, not being aware of which practitioner is responsible for which element of their care, and potentially continued medication use when inappropriate.

### Ambiguities and reservations about roles and responsibilities

There is often confusion about whether primary or secondary care holds ultimate responsibility for some patient monitoring, especially when the specific monitoring requirements and dosage adjustments are quite complex. This can lead to patients falling through the cracks of care, especially when GPs assume that specialists are monitoring or vice versa. There was also a concern that despite GPs having the clinical knowledge to manage these patients in the community, they lack the time to dedicate to the complex administrative tasks and clinical decision-making associated with shared care that would give them the confidence to undertake prescribing in a safe and timely manner.

GPs also have reservations about accepting shared care medications since they are unsure how they might communicate, escalate or repatriate care with the specialist.

### Ambiguous compliance with monitoring requirements

GPs often face dilemmas where they must decide whether to continue prescribing despite outdated monitoring data, particularly for stable patients. GPs often must consider the benefits and risks to the patient based on the information they have available to them to decide whether they continue or interrupt the prescribing of a medication. While this may seem pragmatic, it increases the risk of undetected adverse effects. Also, this flexibility in applying the rules of the monitoring requirements makes it difficult to capture, quantify and evidence the safety. This was discussed when interviewees observed several high-risk medications within the review data that demonstrated low compliance with required monitoring intervals. Interviewees found it challenging to opine whether these indicated safety concerns, since it may have been that the GP had been clinically satisfied with the need and safety of their prescribing decisions.

Interviewees described that some patient-related factors may influence rates of adherence to monitoring appointments. For example, elderly patients and those who are normalised to regular blood testing, for example, methotrexate, might be more diligent in attending monitoring appointments. However, patients with mental health conditions (eg, those on lithium for bipolar disease and patients with ADHD) may be more likely to have barriers in adhering to monitoring practices.

### Inconsistent clinical governance

Interviewees discussed variability in the effectiveness of recall systems for monitoring. In some practices, patients may not be adequately prompted to attend monitoring appointments, leading to delays and increased clinical risk. Also, it was unclear what the process was for recalling patients who had not attended and whether a ‘safety net search’ was routinely undertaken to proactively check for patients with out-of-date test results.

### Medication, equipment and appointment availability

Limited access to appointments and equipment for monitoring, such as for blood tests, can delay essential monitoring, further exacerbating risks. For instance, one interviewee noted a 4-week wait for a blood test, which could critically delay detecting adverse effects and have consequences on the safety of prescribing. Similarly, there were discussions about recent issues with medication supply which could have been better anticipated and managed with input from pharmacy services in the community.

### Communication gaps and ineffective information transfer

Interviewees shared stories and insights about ineffective communication between GP practice and specialists, which can delay decision-making when monitoring results reveal abnormalities. This further endangers patient safety, especially when prompt intervention is required. Repatriation of care back to the specialist appears to be confusing, with minimal identified channels to do this effectively. This leads to delays and disruptions to patient care and the potential for periods where patients do not have the medications they need or remain on medications that put them at risk.

The digital systems are also not appropriately integrated, for example, between the specialist and GP settings, or streamlined eg, between the prescription and monitoring processes, to facilitate transparent patient care, seamless communication and coordination of tasks and safer clinical decision-making.

## Discussion

The shared care model is a complex system requiring coordinated effort from multiple stakeholders. While shared care aims to offer benefits such as improved access to specialist medications in community settings, the current model appears to have numerous challenges that pose a patient safety risk. Key issues include gaps in monitoring requirements, ineffective communication between the specialist and GP setting at all stages (eg, initiating, during and potential repatriation back to specialist care), lack of involvement of the patient (or their representative), clear delineation of roles and responsibilities and lack of robust integrated systems to facilitate data transfer and management for operational and clinical safety and effectiveness.

Reservations about shared care have existed since inception,^3^ where similar concerns concerning poor communication, lack of follow-up and monitoring, poor digital systems to support SCA and ambiguities about the medicolegal responsibilities between the two parties have been raised.^2 3 15 16^ In previous work, the perception of ‘cost-shifting’ from secondary to primary care^3 16^ seems to have disappeared, but our findings demonstrate the emergence of a previously unmentioned concern about the lack of involvement of the patient and/or carer within this model of care.

Our study also highlights how GP pharmacists (with a role in medicines optimisation) and pharmacists in the community (with a role in medicines supply) are not involved in SCA as standard. GP pharmacists have been shown to provide some significant clinical and cost-saving benefits. These include improving Adverse Drug Reaction risk, clinical parameters, medicines adherence, increasing appropriateness of medicines, addressing polypharmacy and reducing healthcare utilisation. Overall, these have demonstrated the potential to enhance safety and medicine use.^17^ The systematic review found no studies where pharmacists had supported GPs in SCA.^18^ Potentially, there is an untapped resource within primary care. However, if the wider system barriers prevail (ie, communication, ambiguities in roles and responsibilities, etc), the involvement of pharmacists is unlikely to improve the safety and effectiveness of SCA.

Our study benefits from a mixed-methods approach where data have been triangulated to provide a rich understanding of the mechanics of SCA, the barriers and probable risks to patient safety. Further useful information could have been to retrospectively collect and review patient safety incidents and/or complaints within the context of SCA and analyse the contributing factors to those reports using human factors methodology. A further enhancement of our work could have been to capture the perspectives of SCA from a secondary care perspective. Specialists and hospital practitioners have previously reported very few concerns with SCA and frustration when their primary care colleagues raise concerns or decline to take over care of the patient. There appears to be a lack of appreciation of the complexities of the landscape and processes within primary care. It would be interesting to investigate this further and explore if and how perceptions within secondary care may change if there was greater awareness of the challenges and patient safety risks of supporting SCA within primary care.

Our findings add to existing limited evidence that paints a cynical picture of SCA since it was introduced. Unfortunately, the issues within the system have not been addressed and appear to be worsened given the acute workforce crisis and pressures of demand within primary care. This increases the patient safety risk and therefore SCA in its current form appears to be in significant need of some attention.

## Conclusions

This study highlights the most current concerns and significant challenges in the implementation of SCAs, including gaps in monitoring adherence, unclear role delineation, inadequate patient involvement and systemic communication failures. These issues pose substantial risks to patient safety, particularly for high-risk medications. Addressing these barriers through improved communication frameworks, robust patient engagement strategies and integrated digital solutions is essential for the sustainability and safety of SCAs. The findings provide critical insights for policymakers, healthcare leaders and practitioners to refine shared care practices and enhance their effectiveness in delivering seamless, safe and patient-centred care.

## Supplementary material

10.1136/bmjoq-2025-003491 online supplemental file 1

## Data Availability

Data are available on reasonable request.
