# Supplementary material for: Specialist medication monitoring and prescribing in primary care: case study of shared care agreements in Northern England, UK
Source: BMJ Open Qual. 2025 Nov 19;14(4):e003491. doi: 10.1136/bmjoq-2025-003491 (PMC12636899; doi:10.1136/bmjoq-2025-003491)
Supplement: online supplemental file 1 [file bmjoq-14-4-s001.docx]

**Appendix 1**: **Audit outline**

**Background**

There are increasing shared care agreements being requested and established. Each medicine within a share care agreement is accompanied with its own monitoring and prescribing thresholds and criteria. In primary care, the monitoring of these shared care drugs and subsequent prescribing is the responsibility of the primary care team. There is a potential that not all criteria are being met with regards to monitoring within the necessary timeframes and actioning appropriately and safely on biochemical results.

**Aim**

The audit aims to investigate the size of this issue with the intention to address this.

**Target for improvement**

The target for improvement will be ensuring that the monitoring criteria and thresholds are being met for shared care medications within primary care.

**Data collection**

- Shared care drugs of interest

| Central Nervous System | Adult ADHD | Methylphenidate | Weight  Heart rate  Blood pressure | Every 6 months |
| --- | --- | --- | --- | --- |
|  |  | Dexamphetamine |  |  |
|  |  | Lisdexamphetamine |  |  |
|  |  | Atomoxetine |  |  |
|  | Motor fluctuations in advanced Parkinson’s | Apomorphine | FBC | Every 6 months |
|  |  |  | Reticulocyte count |  |
|  |  |  | Direct antiglobulin test (Coombs test) |  |
|  | Bipolar/depression | Lithium | Lithium serum levels | Every 3 months |
|  |  |  | Weight/BMI  U&E  Renal function (eGFR)  Thyroid function (TSH)  Calcium | Every 6 months |
|  |  |  | Annual physical health check (BP, lipids, FBG/HBA1Ac, ECG | Every year |
| Cardiovascular/blood | Chronic kidney disease | erythropoietin | Blood pressure (<140/90mmHg) | Monthly |
|  |  |  | Haemoglobin | Every 3 months |
|  |  |  | Ferritin | (as advised by anaemia team) |
| immunosuppression | Rheumatoid arthritis | tocilizumab | Neutrophils | IV: every 4 weeks  SC: every 3 months  If neutrophils <1 contact NUTH DMARD service |
|  |  |  | Liver enzymes (ALT/AST) | As per neutrophils  If ALT/AST rise 3xUNL contact NUTH DMARD service |
|  |  |  | Lipids | Every 6months |

Any other medications that are flagged as being those prescribed under shared care agreements.

Data required from retrospective patient records:

- Date of required test for monitoring, date of actual test, any actions that were taken
- Dates of medication prescribing

**Appendix 2: Shared care medications and monitoring requirements**

| **Medication** | **Monitoring requirements** | **Monitoring intervals** |
| --- | --- | --- |
| Azathioprine | FBC, LFT and U&Es every 3 months | Every 3 months |
| Denosomab | Calcium levels | Every 6 months |
| Lithium | Lithium serum levels | Every 3 months |
|  | Weight/BMI  U&E  Renal function (eGFR)  Thyroid function (TSH)  Calcium | Every 6 months |
|  | Annual physical health check (BP, lipids, FBG/HBA1Ac, ECG | Annually |
| Hydroxychloroquine | No routine laboratory monitoring | |
| Sulphasalazine |  |  |
| Leflunomide | FBC, U&Es, eGFR, creatinine, Alanine aminotransferase (ALT) and albumin | Every 3months |
| Methotrexate | FBC, Creatinine, ALT and albumin | Every 3 months |
| Dexamphetamine  Lisdexamphetamine  Methylphenidate  Atomoxetine | Weight  Heart rate  Blood pressure | Every 6 months |
| Dronedarone | Liver function test and ECG | Every 12months |
| Amiodarone | FBC, U&Es, LFTs, TFTs | Every 6 months |
|  | ECG | Every 12months |
| Mycophenolate | FBC, Creatinine, ALT and/or AST and albumin  ESR and CRP (rheumatology patients only) | Every 3 months |
| Mercaptopurine | FBC, Creatinine, ALT and/or AST and albumin  ESR and CRP (rheumatology patients only) | Every 3 months |

**Appendix 3: Qualitative themes and illustrative quotes**

| **Themes** | **Illustrative quotes** |
| --- | --- |
| Absence of patient input and involvement in shared care | ‘Sometimes unfortunately, shared care agreements pass back and forth, and sometimes they [GPs] decide, actually we're no longer going to do this anymore. So then patients become a little confused as to who's responsible for prescribing their medicines and which pharmacy am I meant to get it from. That happens quite a few times in practice…’  ‘Sadly not at all [patient involvement]. All that happens is they [patients] get really annoyed that, oh, I was told that I had to start methotrexate. And where's my script like, you know, the clinic said that I needed to start it. And you've not done it yet.’  ‘All of a sudden you use this very high risk medicine with an awful lot of other side effects and a lot of monitoring. It's a lot more confusing and actually I think sometimes it is equipping the patient with all that information to decide. Is it something they actually want to do? Forgetting the whole coordination, liaison with services, but actually to fully understand what that medicine is, all the risks and side effects.’  ‘There's a sheet for GPs to sign it, whereas I do worry that the patient doesn't get all the appropriate counselling time to decide.’ |
| Ambiguities and reservations about roles and responsibilities | ‘But normally the push back from the GP is because they feel like they either don't have the capacity or they don't have the correct skill sets to be able to continually monitor those patients’  ‘Specialist assumes the GP agrees to it and there's a lack of discussion between all the parties in regards to who's responsible for what exactly. Yeah, I said there needs to be clear communication from the start. I think the consent from the GP must be written down. Can't just assume shared care because. So a lot of these medicines are high risk and very specialised.’  ‘…the eye exam or checking about heart failure, it's often around the bloods and urine dips.... But again, it's sort of whose responsibility is that?’  ‘The supply of these medicines and the prescribing of them…it needs to be very clear to the GP. They're not specialists … in cardiology with dronaderone, for example, they, they need to be told what they're looking out for. It needs to be clear guidance.’  ‘So a lot of these medicines are high risk and and very specialised. And yeah, if there's a lack of discussion between who's responsible for what, and especially in the initial stages, then it's a huge area for disaster. If it's not done correctly,’  ‘So I think that a lot of understanding from both people is needed so everyone knows what the other person's doing. So I suppose ambiguity when it comes to shared care is a big issue.’ |
| Ambiguous compliance with monitoring requirements | ‘Understanding from the GP staff as to … monitoring requirement should be for each group, yeah, I think if the monitoring is not done correctly then it's not safe to do the shared care agreement.’  ‘We used to insist on three monthly bloods. And then COVID came around and it was six months and then it was nine months. And then kind of well, things are now settling down a little bit. Do we need to go back to three months or do we can we keep it at nine months? And I think the jury's still out on what is it actually.’  ‘Just the nature of the condition and the problem by not prescribing the lithium. Or do you just take a risk and keep going with it? Personally I don't think three monthly lithium levels are that useful when it comes to monitoring patients who are stable.’  ‘What a GP or the shared care agreement might consider a kind of warning blood result might be not an issue for the specialist and it's takes that specialist kind of knowing the patient, knowing the condition, to be able to make that call. But I think sometimes things are escalated or not escalated.’ |
| Inconsistent clinical governance | ‘I mean, I actually, I just worry that although it's called shared care and there is some monitoring actually, because no one's taken the full overall responsibility, no one's quite over overseeing it’  ‘We have one person, an admin that …run these searches, look for the people not attending, remind them to come in. But …it's costly for primary care to employ administrators to check on that…Even so, we can't guarantee that actually…you then always have that seamless care through the other side, so, you know, actually it's quite a lot of increased resource in primary care to do it.’  ‘I guess like a practise could have like 20 different shared care agreements going on at one time and as long as they have those resources available for each one to be able to check what the safe levels of kidney function or the electrolyte results are then it’s fine.’  ‘…yeah, I think if the monitoring is not done correctly then it's it's not safe to do the shared care agreement.’  ‘And it [medication] was started by consultants who are now retired and their caseloads have been shared out. And lots of these patients have never met any of the cardiologists who currently work for the trust. So I think that's a real risk because, yeah, there's always going to be people who don't show up for bloods and that sort of thing. But patients who stay on these dangerous drugs for a long period of time without a proper review, that’s not good.’  ‘But I think historically there'll be lots of patients who have just been lost, have moved around, moved practises, moved hospitals, that sort of thing. Their condition is well controlled and they're just not having that critical eye because the specialists are only seeing the patients who are actively have a problem that if you're kind of getting on with things, they're not going to actively hunt you down most of the time. Unfortunately, just because of their workload.’ |
| Medication, equipment, and appointment availability | ‘I guess the ADHD medicines as well, they're on there and obviously there's always an ongoing supply issue with ADHD medications.’  ‘To take bloods, to do an ECG and have someone on hand to interpret an ECG and all that sort of stuff is far more complicated than doing a high heart rate and blood pressure. So it's having the right people in the right place and can communicate to the pharmacy, and just share that information with the patient's GP’  ‘I don't know if you look into this kind of community pharmacy, have they got their systems in place to get hold of the right drugs, dispense them safely, kind of. Are they available because of certain drugs aren't available to your pharmacy?’ |
| Communication gaps and ineffective information transfer | ‘But the worry is we're getting more shared care without necessarily the paperwork or the advice, the guidance around it. But I would say on a weekly basis, we're having problems feeding back into the hospital issues with shared care.’  ‘I actually worry about the dosing because I think there are delays in the communication back and forth and I think that actually what the hospital believes the patients are receiving might not be the same as actually what the patients are receiving and that could be delay in the communication between the services.’  ‘With the hospital, there's often never a port of call. There's no one in the hospital to actually approach… Always someone accessible that you can contact.’  ‘the only thing to say on that digital recording system that brings medication, monitoring information together is actually the hard thing in our region…System doesn't always work, but yeah, ideally you want the results. You'd want the ideal system that we're all using the same thing, and we can all see what everyone's done and where we're at’  ‘So I think that's probably patients are lost at follow up in secondary care because the GP is not going to start adjusting doses of methotrexate or amiodarone.’  ‘Yeah, the communication kind of knowing what to do when things go wrong, making sure that you have routes to escalate problems and that specialists are still happy for these things to be continued.’ |
